# Enhanced Digital Light Processing-Based One-Step 3-Dimensional Printing of Multifunctional Magnetic Soft Robot

**DOI:** 10.34133/cbsystems.0215

**Published:** 2025-02-26

**Authors:** Zhaoxin Li, Ding Weng, Lei Chen, Yuan Ma, Zili Wang, Jiadao Wang

**Affiliations:** State Key Laboratory of Tribology in Advanced Equipment, Department of Mechanical Engineering, Tsinghua University, Beijing 100084, China.

## Abstract

Soft structures driven by magnetic fields exhibit the characteristics of being unencumbered and rapidly responsive, enabling the fabrication of various soft robots according to specific requirements. However, soft structures made from a single magnetic material cannot meet the multifunctional demands of practical scenarios, necessitating the development of soft robot fabrication technologies with composite structures of diverse materials. A novel enhanced digital light processing (DLP) 3-dimensional (3D) printing technology has been developed, capable of printing composite magnetic structures with different materials in a single step. Furthermore, a soft robot with a hard magnetic material–superparamagnetic material composite was designed and printed, demonstrating its thermal effect under high-frequency magnetic fields and the editability of the magnetic domains of the hard magnetic material. The robot exhibits a range of locomotive behaviors, including crawling, rolling, and swimming. Under the influence of a 1-Hz actuation magnetic field, the normalized velocities for these modes of motion are recorded as 0.31 body length per second for crawling, 1.88 body length per second for rolling, and 0.14 body length per second for swimming. The robot has demonstrated its capacity to navigate uneven terrain, surmount barriers, and engage in directed locomotion, along with the ability to capture and transport objects. Additionally, it has showcased swimming capabilities within environments characterized by low Reynolds numbers and high fluid viscosities, findings that corroborate simulation analyses. The multimaterial 3D printing technology introduced in this research presents extensive potential for the design and manufacturing of multifunctional soft robots.

## Introduction

With the advancement of micro-nano fabrication technologies, a variety of wireless-driven soft microstructures have emerged [1]. These structures, owing to their rapid and reversible motion and noncontact control, perform uniquely in narrow and liquid environments, with applications in soft sensors [2–4], micromanipulation [5,6], drug delivery [7–9], magnetothermal therapy [10–12], and microfluidics [13–15]. Among driving methods like light [16–18], thermal [19–21], and pH stimuli [22,23], magnetic field actuation stands out due to its rapid response, noninvasiveness, biocompatibility, and capability for remote control through the human body [24].

To mitigate the substantial discomfort caused by traditional medical devices like gastroscopes and colonoscopes, numerous robots leveraging magnetically driven soft structures have been developed to replicate certain functionalities of these instruments [25–27]. For instance, magnetically actuated capsule endoscopes facilitate noninvasive examinations of the upper gastrointestinal tract, offering greater patient tolerance and acceptance compared to conventional gastroscopy [28]. However, traditional medical devices, equipped with wired connections, can incorporate various auxiliary functional components, such as cameras, light sources, balloons, biopsy forceps, and water delivery systems [29–31]. In contrast, single-material magnetically driven soft structures exhibit limited functionality, which hinders their ability to fully replace traditional medical devices. Consequently, the incorporation of new materials into magnetically actuated soft structures to create soft robots with composite architectures, thereby broadening their range of applications, represents a key direction for the future advancement of soft robotics [32–35].

Currently, various methods are available for fabricating 2-dimensional (2D) or 3D magnetic structures, as summarized in Table. However, traditional processes like mold-assisted forming [36–38] and ultraviolet (UV) lithography [39–41] are restricted by mold shapes and material types, posing challenges in fabricating complex magnetic structures. These methods demand uniform composition throughout the structure, complicating the creation of multifunctional magnetic structures with multiple materials. While multistep assembly and material bonding techniques can combine different materials for various applications, they struggle with ensuring the size range and precision of the fabricated structures [42].

**Table.** Summary of preparation methods for magnetically actuated soft structures

Preparation method	Materials for preparation	Capability for multimaterial one-step preparation	Shape of structure	Size range (mm)
Mold-assisted thermoset silicone rubber molding method [60]	Silicone elastomer (Ecoflex 00-10) + 5 μm NdFeB particle	No	3D	1–100
Mold-assisted UV curing method [61]	UV resin (GC3D-EBE) + 5 μm NdFeB particle	No	2D	0.1–10
Manual assembly of oriented elements [42]	Silicone elastomer (Ecoflex 00-50) + 5 μm NdFeB particle	No. But it can be assembled with the aid of assistant jigs.	3D	0.1–10
An optofluidic maskless lithography system [62]	Superparamagnetic colloidal nanocrystal clusters (Fe_3_O_4_ CNCs) + a photocurable resin monomer solution	No	2D	0.01–1
Extruded light-curing 3D printing method [49]	UV resin (UV electro 225-1) + 5 μm NdFeB particle	No	2D	0.1–100
Extruded silicone rubber 3D printing method [63]	Silicone elastomer (SE 1700 + Ecoflex 00-30) + 5 μm NdFeB particle	No	3D	1–100
Two-photon induced photopolymerization [64]	SU-8 photoresist + Ni/Ti bilayers (physical vapor deposition)	Yes	3D	0.001–0.1
Microcontinuous liquid phase interface method [65]	UV resin (CN981 NS + TPGDA + PEGDA) + NdFeB:Fe_3_O_4_ = 1:1	No. But has potential.	3D	0.01–10
Circulating vat photopolymerization method [47]	UV resin (XYZ printing flexible resin B1) + 3–6 μm SrFe_12_O_19_ particle	No	3D	0.1–100
Digital light processing (DLP) [52]	UV resin (Elastic 50 A) + 5 μm NdFeB particle	Yes	3D	0.1–100
Digital light processing (DLP) [66]	UV resin (PEG-PPG + Genomer 1122 + CTFA) + 200 nm Fe_3_O_4_ particle	No. But has potential.	3D	0.1–100
This work	UV resin (H-04) + 5 μm NdFeB particle	Yes	3D	0.1–100

3D printing technology presents substantial advantages in fabricating magnetic structures, with DLP exhibiting superior precision in the 0.01- to 100-mm size range compared to traditional mold-forming techniques [43]. DLP also outperforms UV lithography in fabrication efficiency and can produce complex 3D structures in a single step. However, the literature reveals persistent challenges in 3D printing magnetic structures. In DLP, commonly used magnetic materials include Fe_3_O_4_ [44], NdFeB [45,46], SrFe_12_O_19_ [47], and Fe [48]. Soft magnetic materials like Fe, despite their high permeability and ease of magnetization, provide weak driving force. Hard magnetic materials, such as NdFeB, exhibit poor stability in resin, often leading to agglomeration [47]. In the process of direct ink writing (DIW), the use of a soft thermosetting silicone rubber matrix limits the ability to print complex hollow structures, and the printing resolution is constrained by the nozzle diameter [49]. Additionally, there is currently no literature reporting the ability of DIW to print multimaterial composite structures in a single step.

The “Capability for multimaterial one-step preparation” column indicates whether the preparation process can accommodate different materials without interruption or structural deformation. The “Shape of structure” column specifies the dimensionality of the exhibited structures. The “Size range” column outlines the size range of the exhibited structures.

Recent research has developed techniques to co-print structures containing both NdFeB-infused soft resin and conventional soft resin [50]. Other studies have demonstrated the combined printing of rigid and soft resins both infused with the same magnetic material [39]. Additional work reports on the layered printing of superparamagnetic particles with carbon nanotubes, enabling dual magnetothermal actuation in the resulting materials [51]. Although the concept of co-printing different magnetic materials has been proposed, no substantial progress or detailed findings have yet been reported [52]. Enhancing the diversity of materials within a structural composition is instrumental in broadening its functional spectrum. The inclusion of materials that exhibit thermal [53], electrical [54], and optical [55] responsiveness can confer a myriad of innovative attributes upon the structure.

To bridge the gap in one-step 3D printing of multimaterial magnetically actuated soft structures, this study introduces an advanced DLP technique. This method enables the fabrication of composite magnetic structures comprising different materials in a single printing process. Utilizing this technique, we fabricated various composite structures, including magnetic soft–hard material composites, gradient composites with different concentrations of magnetic materials, and hard magnetic-superparamagnetic composites. A magnetically driven soft robot was designed using the hard magnetic-superparamagnetic composite structure to demonstrate the technique. This robot validated the thermal effects of superparamagnetic materials under high-frequency magnetic fields and the editability of magnetic domains in hard magnetic materials. By designing the robot’s shape and magnetic domain distribution, we verified its dual-mode ground movement, obstacle-crossing ability, movement on rough surfaces, and capability to grasp and adhere to objects. Additionally, through multiphysics coupling simulations and comparative experiments, the robot’s swimming ability in high-viscosity liquid environments was studied.

## Materials and Methods

### Materials

The polymer component of the magnetic resin is composed of UV curable resin (405 nm, H04, Hesu Light Curing Material Co. Ltd., China), mixed with fumed silica (AEROSIL R972, Evonik Degussa, Germany), a high-molecular-weight dispersant (BYK 163, BYK-Chemie, Germany), and a rheological additive (Disparlon 6900-20X, Kusumoto Chemicals, Japan). The magnetic materials in the resin can be either NdFeB particles (*D*50 = 5 μm, Magnequench, China) or Fe_3_O_4_ particles (*D*50 = 200 nm, Shanghai Naiou Nano Technology Co. Ltd., China). To prepare the polymer component, 1 wt % of the high-molecular-weight dispersant and 3 wt % of the rheological additive are added to the UV curable resin. The mixture is stirred using a magnetic stirrer at 60 °C and 700 rpm for 4 h. After filtration and cooling, the magnetic materials are added to the polymer according to the required proportion (up to 20 wt % NdFeB or up to 10 wt % Fe_3_O_4_) and stirred mechanically at 20 °C for 4 h. The mixture is then vacuumed for 30 min to obtain the final magnetic photopolymer resin.

### Fabrication

The 3D printing apparatus used was modified from a commercial DLP device (Mono 2, Anycubic Technology Co. Ltd., China). The resin tank’s bottom release film was replaced with an advanced composite film (ACF) purchased from Shenzhen 3DSWAY Technology Co. Ltd., China. To prevent potential issues with sample adhesion during the printing of high-concentration magnetic powder samples, a preliminary layer of transparent resin was printed as a transitional layer to improve the success rate of the print. No specific treatment of the sample is required when switching resin tanks for printing composite structures, but it is recommended to wipe off excess uncured resin from the sample’s surface to prevent cross-contamination of the resin tank. After printing, the sample needs to be soaked in alcohol for 1 min to remove uncured resin residues, followed by air drying and secondary curing under 405-nm UV light for 5 min. Due to potential deformation from residual stresses in thin-walled structures, the sample should be left to rest for 7 d to allow the residual stress to dissipate.

### Measurements

The surface morphology and elemental analysis of the samples were characterized using a field-emission scanning electron microscope (SU8220, Hitachi Ltd., Japan). Magnetic properties of the printed structures were quantified using a high-sensitivity magnetic measurement system (SQUID-VSM, Quantum Design Inc., USA). The yield stress and fracture strain of the printed structures were measured using an electronic universal testing machine (AG-IC20KN, Shimadzu Corporation, Japan). Magnetization of the soft robots was achieved with a custom-built electromagnet, with a magnetic gap of 8 mm and a magnetic field strength of 1.2 T. The thermal effects of high-frequency magnetic fields on the soft robots were evaluated using a custom-built electromagnetic induction heating device, with a maximum induction heating power of 1,500 W.

### Magnetic field

In the comprehensive evaluation of a soft robot’s capabilities for ground locomotion, obstacle traversal, and object manipulation, NdFeB permanent magnets were employed. During ground movement, the robot was subjected to a peak driving magnetic field of 20 mT. For swimming tests, a custom Helmholtz coil was utilized to generate the magnetic field, with control provided by a signal generator (FY6300, FEELTECH Technology Co. Ltd., China) and a power amplifier (FPA100, FEELTECH Technology Co. Ltd., China). The peak magnetic field experienced by the robot during swimming was 10 mT.

## Results and Discussion

This section introduces the innovative aspects and implementation principles of a multimaterial one-step 3D printing technology for magnetically driven soft structures. It includes mechanical and magnetic characterizations of the 3D-printed structures, along with demonstrations of sample applications. The design and validation of soft robots composed of hard magnetic materials and superparamagnetic materials are then detailed, focusing on the magnetic domain distribution and the thermal effects of the superparamagnetic material. The ground mobility of these soft robots is evaluated, showcasing their abilities to overcome obstacles and capture and transport objects. Furthermore, the swimming capabilities of the robots in liquid environments are examined, and their swimming postures are analyzed using multiphysics coupled simulations.

### Implementation of 3D printing technology

The magnetic photosensitive resin developed in this study is created by embedding magnetic particles within a soft polymer matrix. Depending on the specific printing requirements, the magnetic particles used are primarily NdFeB hard magnetic particles with an average diameter of 5 μm, and Fe_3_O_4_ superparamagnetic particles with an average diameter of 200 nm. Printing can also be performed without adding magnetic particles. The soft polymer matrix includes a photosensitive resin enhanced with fumed silica, high-molecular-weight dispersants, and rheological additives. Dispersing high concentrations of magnetic powder in the resin is challenging; NdFeB hard magnetic particles, due to their large size of 5 μm, tend to agglomerate and settle, while Fe_3_O_4_ nanoparticles strongly absorb UV light, reducing the curing depth of the resin by 30% under UV exposure [47]. The addition of high-molecular-weight dispersants, rheological additives, and fumed silica improves the dispersibility of the magnetic particles. NdFeB at the micrometer scale, renowned for its elevated remanence, is a prominent hard magnetic material in contemporary research endeavors and is anticipated to see expanded applications within the digital light processing (DLP) printing domain. Fe_3_O_4_ nanoparticles are broadly utilized for ascertaining the thermal effects imparted by superparamagnetic particles when subjected to high-frequency magnetic fields, and this material is also increasingly featured in DLP printing technologies. However, the determination of its concentration is governed by the constraints inherent to manufacturing methodologies and the stringent requirements for modulating the magnitude of thermal effects.

**Fig. 1.**
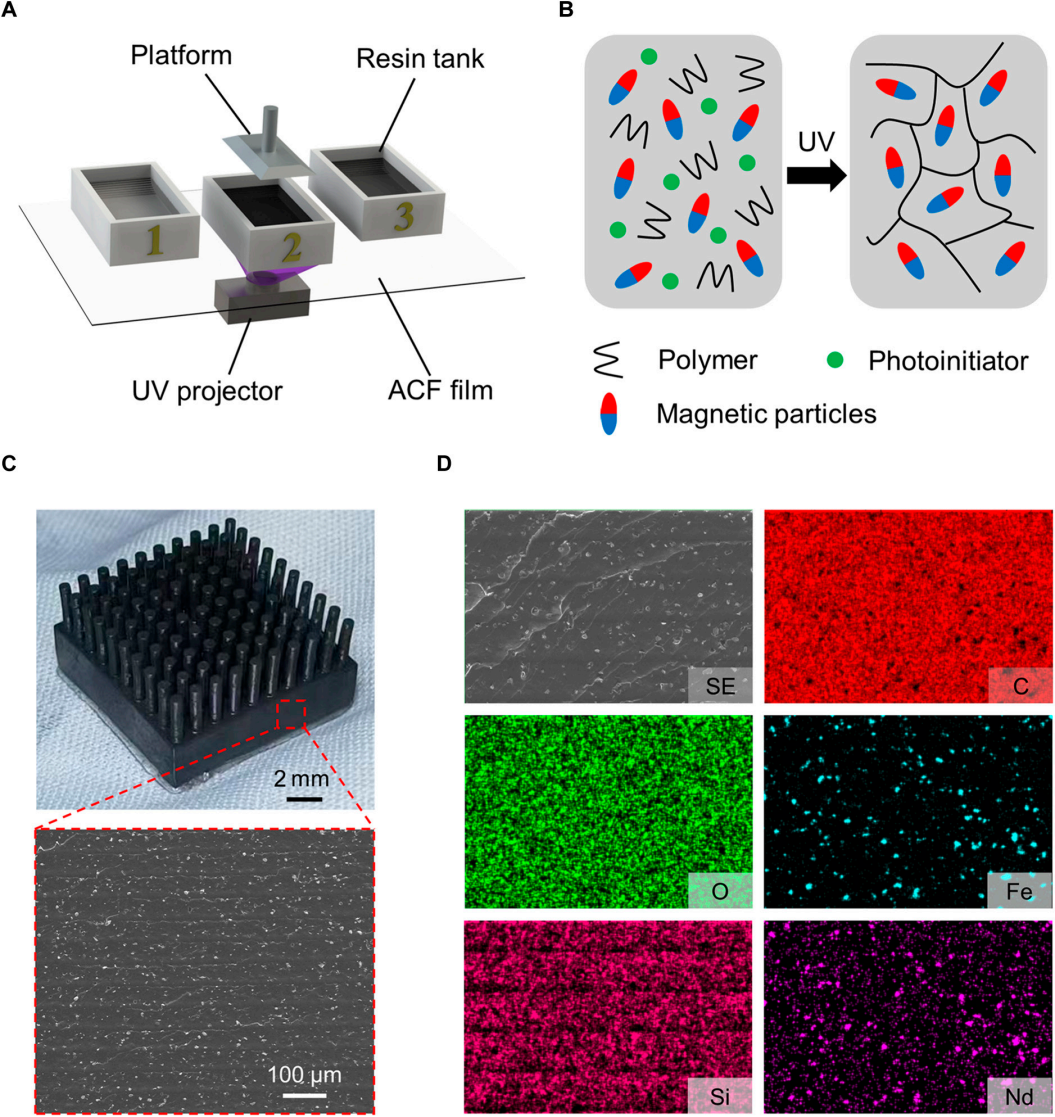
Schematic illustration and SEM images of the multimaterial one-step printing process and the printed samples. (A) Schematic of the multimaterial one-step printing process, demonstrating the sequential switching of 3 different resin tanks during printing to achieve composite structures in a single step. (B) Illustration of the curing process of resin containing magnetic particles under UV light. (C) SEM image of the cross-section of a printed sample, indicating uniform layer height and stable printing. (D) EDS image of the printed sample, depicting the elemental distribution across the sample’s cross-section.

Additionally, printing resins with high concentrations of magnetic materials requires modifications to the DLP printer to increase UV illumination power and thereby enhance the printing depth. However, increasing UV intensity causes the bottom resin layer to adhere to the release film, preventing the lifting of the printing platform. This issue is resolved by using ACF release film, which has lower adhesion, and reducing the UV curing time. For multimaterial one-step printing, printing can be paused to switch material types by changing the resin tank, allowing for the fabrication of soft structures with different materials at various heights. Different magnetic materials necessitate distinct stable printing parameters; thus, the printer’s control program must be modified to set different parameters for each layer, ensuring stable printing results. During the process of 3D printing magnetic resins, the UV light power is consistently set at 4,200 μW/cm^2^. For resins containing NdFeB, the exposure time for the base layer is configured to 40 s, with a standard exposure time of 30 s for subsequent layers. In the case of resins incorporating Fe_3_O_4_, the base layer exposure time is extended to 45 s, while the standard layer exposure time is 35 s. The layer thickness for all printed structures is set to 50 μm. Considering the increased viscosity of the magnetic resins, the platform lift height after a single print should exceed 8 cm. A schematic diagram of the printing process is shown in Fig. 1A.

The curing mechanisms of various magnetic particles within the resin appear to be similar, implying that they do not engage in the crosslinking reactions during the photopolymerization process but are uniformly embedded within the resin matrix, as depicted in Fig. 1B. This assertion is supported by the scanning electron microscopy (SEM) image shown in Fig. 1C. Additionally, SEM images reveal that the interlayer thickness of the 3D-printed structures consistently measures around the predetermined 50 μm, with a deviation of no more than 1%, indicating excellent printing stability. The distribution of magnetic particles within the resin does not show marked signs of agglomeration. As illustrated in Fig. 1D, energy-dispersive spectroscopy (EDS) mapping images further corroborate the uniform distribution of magnetic particles within the samples. Although this study only investigated NdFeB particles with a diameter of 5 μm and Fe_3_O_4_ particles with a diameter of 200 nm, neither type of particle underwent surface modification or participated in crosslinking reactions. This suggests that the technique has promising potential for extension to other functional particles as well. Enhanced magnetic resins, incorporating rheological modifiers, demonstrate notably improved stability when compared to standard resins, as illustrated in Fig. S2A. Considering that the printing duration typically does not exceed 6 h, these resins maintain the structural integrity by preventing substantial aggregation and sedimentation of magnetic fillers throughout the printing procedure.

### Characterization of 3D-printed structures

**Fig. 2.**
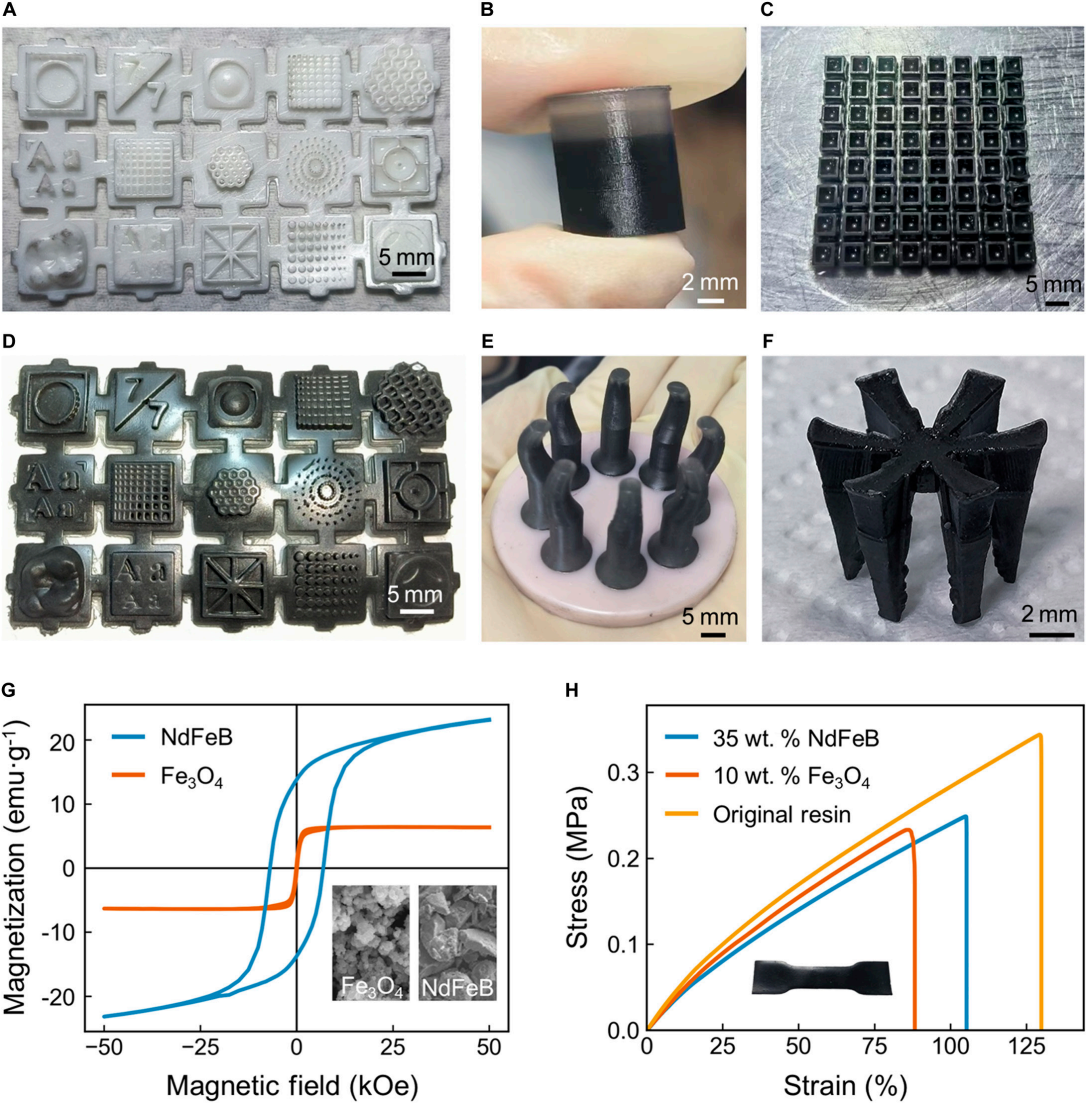
Characterization of 3D-printed samples. (A) Sample printed using standard rigid resin to verify printing accuracy for comparison. (B) Multilayer sample printed with soft resin containing NdFeB particles, where the 6 layers of the cylinder from top to bottom have magnetic particle proportions of 0, 0.5, 1, 5, 10, and 20 wt %. (C) Sample printed with soft resin containing NdFeB particles, with 64 rectangular thin-wall structures of 500-μm wall thickness and 5-mm height printed in one go. (D) Sample printed with soft resin containing NdFeB particles to verify printing accuracy for comparison. (E) One-step printing of a conceptual soft gripper device using both standard rigid resin and soft resin containing NdFeB particles. (F) Combination structure of a cup printed in one step using soft resin containing NdFeB particles and soft resin containing nano-Fe_3_O_4_ particles. (G) Hysteresis loops of soft resin containing 20 wt % NdFeB and soft resin containing 10 wt % Fe_3_O_4_, indicating the hard magnetic properties of NdFeB resin and the superparamagnetic properties of Fe_3_O_4_ resin. (H) Tensile stress–strain curves of soft resin containing 20 wt % NdFeB, soft resin containing 10 wt % Fe_3_O_4_, and the original resin.

To verify the accuracy of 3D-printed structures, both standard rigid resin and magnetically responsive soft resin were used to print identical 3D test models, as illustrated in Fig. 2A and D. The models included cylinders with a minimum diameter of 100 μm, thin-walled structures, support structures, and 3D grid structures. The verification results demonstrated that replacing the resin did not substantially impact printing accuracy, with overall and detailed dimensional errors within acceptable limits (<1%). However, increasing the intensity of UV exposure facilitated the successful printing of high aspect ratio protruding structures while making it more challenging to print high aspect ratio recessed structures. This is because enhanced UV power reduces the precision of the projection light spot for dimensions smaller than 1 mm, leading to larger actual curing areas that easily fill small pores with resin. Moreover, compared to rigid resin, soft resin exhibited more noticeable residual stress after curing with increased UV intensity. The lower elastic modulus caused the structure to curl easily, requiring over a week of air exposure to completely eliminate residual stress. The Shore hardness of the soft resin, which is suitable for stress relief, ranges from 35A to 45A. In contrast, the rigid resin exhibits a higher hardness, with values between 83D and 86D. Notably, the incorporation of NdFeB or Fe_3_O_4_ into both types of resins does not result in a substantial change in Shore hardness.

To verify the accuracy and stability of multimaterial one-step printing, 4 sets of experiments were conducted. First, soft resin containing NdFeB particles was printed with varying proportions of magnetic particles in the cylinder’s 6 layers from top to bottom: 0, 0.5, 1, 5, 10, and 20 wt %, as shown in Fig. 2B. The final printed structures exhibited good consistency in surface flatness, dimensional accuracy, and particle dispersion, confirming that resin with a single magnetic particle composition could print samples with different proportion combinations in one go. Subsequently, the stability of the printing was tested by printing 64 thin-walled rectangular structures with a wall thickness of 500 μm and a height of 5 mm, achieving a success rate of 95.3%, as shown in Fig. 2C. The 3 failed thin-walled structures exhibited slight deformation due to residual stress, which recovered to a rectangular shape after stress release. Furthermore, a conceptual soft gripping device was printed using both standard rigid resin and soft resin containing NdFeB particles in one step, as shown in Fig. 2E. The base was a 5-cm-diameter rigid platform with 8 symmetrically distributed soft gripping claws on the upper part. The rigid base could be pre-printed with holes for connection and alignment with existing commercial robotic arms with high precision [56]. Unlike purely soft gripping structures that rely on post-assembly to connect to robotic arms for actuation, the integrated printing of soft and rigid structures enhanced connection precision and strength, expanding the application prospects of soft structures in combination with other technologies. Finally, a combination structure of hard magnetic material and superparamagnetic material was printed using soft resin containing NdFeB particles and soft resin containing Fe_3_O_4_ nanoparticles in one step, as shown in Fig. 2F. The hard magnetic structure retains high remanence at room temperature [57], allowing magnetic domain editing through magnetization and controlling structural deformation and movement via external magnetic fields. The superparamagnetic structure, with zero remanence at room temperature, does not interfere with the magnetic domains of the hard magnetic structure. Additionally, the superparamagnetic structure can generate thermal effects in high-frequency magnetic fields [58], enabling remote structure heating with potential applications in cancer hyperthermia and drug release [59].

To verify that the printed structures retain the inherent properties of the magnetic particles, we measured the hysteresis loops of the printed structures using a high-sensitivity vibrating sample magnetometer. The results showed high remanence characteristics for NdFeB particles and negligible remanence for nano-Fe_3_O_4_ particles, as depicted in Fig. 2G. Additionally, SEM images of the original states of the 2 types of particles were captured. Nano-Fe_3_O_4_ particles were observed as agglomerated spherical particles, while NdFeB particles appeared as irregular fragments. To assess the strength of the printed structures, tensile tests were conducted on structures with both types of particles and a soft resin structure without magnetic particles, as shown in Fig. 2H. The structures containing magnetic particles exhibited greater softness compared to the structure without particles, with the Young’s modulus of the NdFeB structure at a 20 wt % ratio being lower than that of the Fe_3_O_4_ structure at a 10 wt % ratio. This property not only facilitates deformation in the magnetic field but also reduces the yield stress and fracture strain of the material. The fracture strain of the Fe_3_O_4_-containing structure was lower than that of the NdFeB structure, potentially due to the higher UV absorption of Fe_3_O_4_ [47]. Consequently, the manually reduced curing time during the curing process may have had a more substantial impact on the mechanical strength of the Fe_3_O_4_-containing structures, leading to less complete and uniform crosslinking and earlier fracture of these structures. Incorporating a high concentration of magnetic particles into resin typically diminishes the interfacial strength, and the agglomeration and sedimentation of particles can lead to serious multimaterial interface issues. The connection strength at the interface of the structure was tested and found to be consistent with the tensile test results anticipated in the manuscript. Resin containing Fe_3_O_4_ could withstand a lower maximum tensile stress compared to that containing NdFeB, hence its earlier fracture occurrence. Among all the samples tested, no interfacial fractures were observed, as depicted in Fig. S2B.

### 3D-printed hard magnetic-superparamagnetic soft robots

The soft robot, integrating hard magnetic and superparamagnetic materials, was fabricated using a one-step printing technique as previously outlined. A central symmetrical design with 6 soft robotic limbs is featured, with the uppermost and lowermost layers being transparent soft resin devoid of magnetic particles. Sandwiched between these are layers of resin embedded with hard magnetic materials and superparamagnetic materials, respectively, each with a uniform thickness of 500 μm, while the transparent resin layers are 50 μm thick. To ensure robust adhesion to the platform and prevent detachment during the printing process, the curing duration for the base transparent resin layer was extended to twice that of the standard layer. This adjustment resulted in an increased curing area for the base layer, with an outer diameter approximately 105% larger than the standard 10-mm layer.

**Fig. 3.**
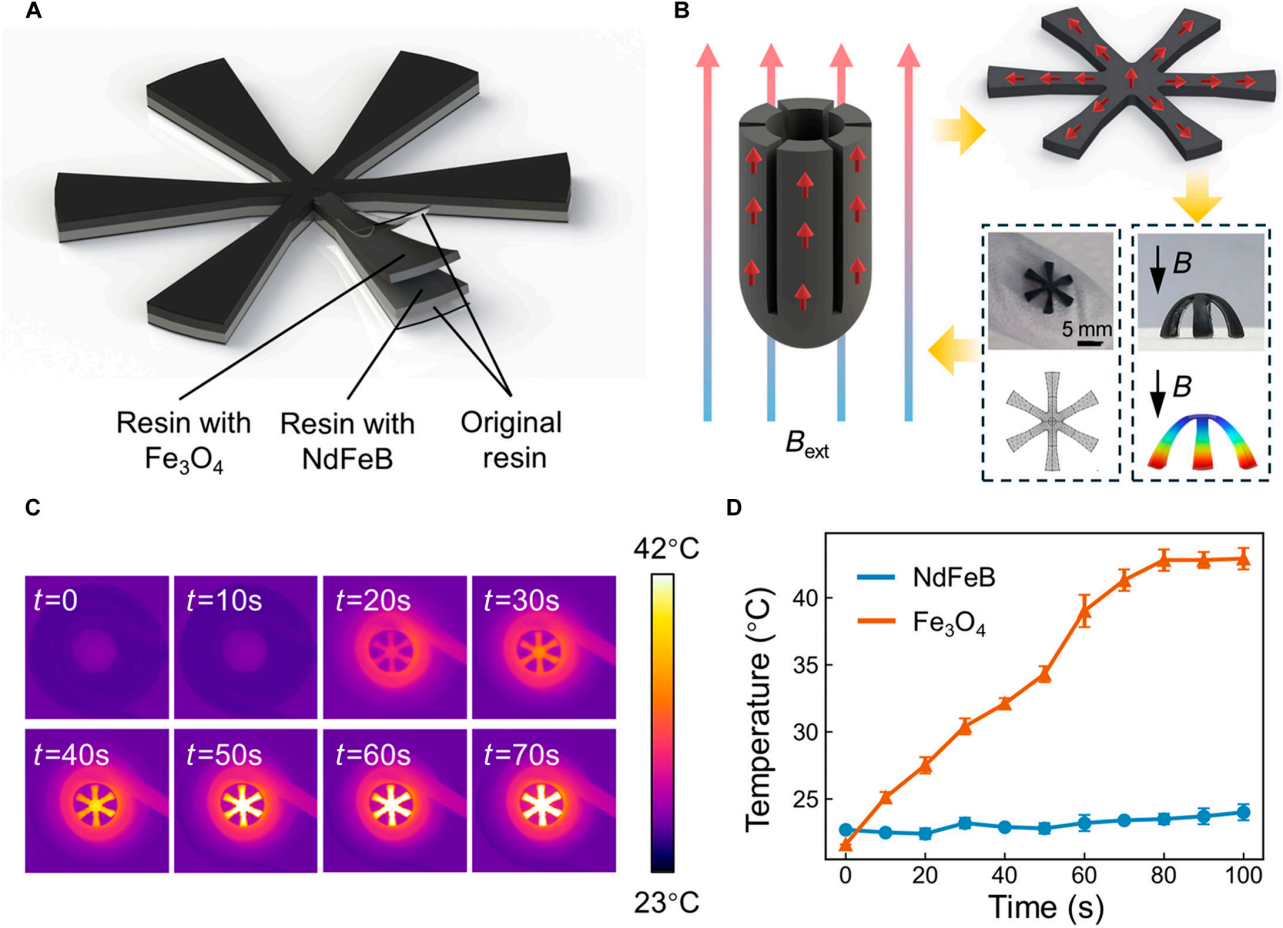
Schematic of magnetization and thermal effect characterization of hard magnetic material–superparamagnetic material soft Robot. (A) Schematic of the hard magnetic material–superparamagnetic material soft robot, with the robot divided into 4 structural layers for printing from top to bottom. The robot features a jellyfish-like structure with 6 soft arms, symmetrically arranged around a central axis. (B) Schematic of the robot’s magnetization process. On the left, the process of re-editing the robot’s magnetic domains using a mold is shown. The upper right shows the distribution of residual magnetic flux density after magnetization, while the lower right presents a comparison between experimental results and simulations of the robot’s deformation under a magnetic field. (C) Infrared imaging of the robot under high-frequency induction heating. The robot’s temperature rises from room temperature to 42 °C within 70 s. (D) Temperature variation of a circular magnetic structure with the same outer diameter and thickness as the robot within a high-frequency induction coil. The thermal effect of the Fe_3_O_4_ soft structure is consistent with that of the robot, whereas the NdFeB structure does not exhibit a noticeable thermal effect.

The robot’s shape was stabilized with the aid of a mold, and its magnetic domains were reprogrammed using an external magnetic field of 1.2 T. The magnetic domain distribution before and after magnetization is detailed in Fig. 3B. COMSOL Multiphysics software was employed to simulate the magnetization process, revealing that the robot’s overall deformation under the external magnetic field correlated well with experimental data. However, discrepancies were observed between the experimental and simulated deformation curvatures of the robot’s arms, attributed to residual stresses within the base layer and frictional forces at the ground interface. In order to prevent residual stress from adversely affecting the deformation of the structure, an annealing treatment is essential. Additionally, the structure should be left to rest in a well-lit environment for a period of 72 h to ensure that any residual stress is effectively eliminated.

The thermal response of the robot’s superparamagnetic material layer was assessed using a high-frequency electromagnetic induction heating apparatus. Within a 70-s interval, the robot’s temperature escalated from ambient to 42 °C, as illustrated in Fig. 3C and Movie S1. This property underscores the robot’s applicability in cancer thermotherapy and drug delivery, harnessing heat generated by magnetic fields. To ascertain whether this phenomenon is attributed to the superparamagnetic characteristics of nanoscale Fe_3_O_4_, a comparative experiment was conducted. A circular magnetic structure, identical in dimensions to the robot, was 3D-printed and subjected to the induction heating coil. The findings, presented in Fig. 3D, indicate that the structure augmented with nanoscale Fe_3_O_4_ mirrored the robot’s thermal response, in contrast to the structure with NdFeB, which lacked a pronounced heating effect. The induction heating coil, constructed from a hollow copper tube, was designed to mitigate temperature through a continuous flow of cooling water. Nonetheless, due to its intrinsic resistance, some residual heat generation was inevitable. The NdFeB-embedded structure registered a modest temperature rise of about 2 °C over 100 s, potentially a consequence of the coil’s inherent thermal generation.

To verify the robustness and reliability of robotic cantilever beam structures with embedded magnetic particles, their deformation capabilities under harsh environmental conditions were scrutinized. Experiments included exposure to an acidic environment with pH 1.5, an alkaline environment with pH 14, and a high-temperature environment at 100 °C. For each cycle, the cantilever beam was immersed in the designated environment for 1 h before being removed. After each cycle, the beam’s deflection angle was measured under a uniform magnetic field, with the results normalized for consistency. The findings revealed that after 10 cycles, the structure maintained 97% of its deformability in the acidic environment, 93% in the alkaline environment, and 62% in the high-temperature environment. These results not only underscore the structure’s resilience to acid and alkali but also highlight the demagnetization of NdFeB at elevated temperatures. The graphical representation of the experimental outcomes is depicted in Fig. S3.

### Terrestrial locomotion capability of soft robots

**Fig. 4.**
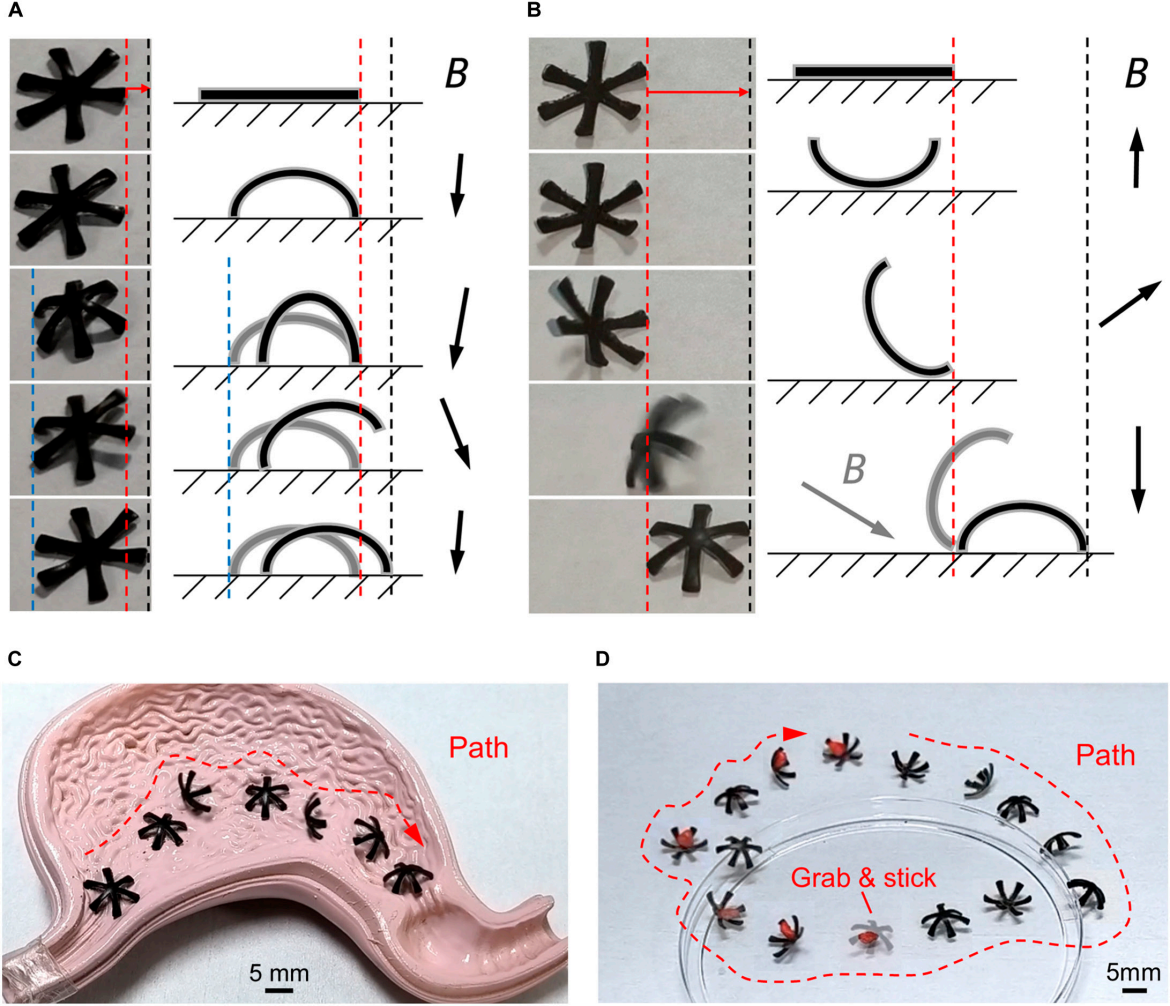
Robotic locomotion and functional validation on a plane. (A) Experimental results and schematic of the robot’s crawling posture. The blue line indicates the robot’s starting position on the left, the red line represents the robot’s starting position on the right, and the black line denotes the robot’s termination position on the right. The black arrow signifies the direction of the magnetic field. The normalized speed of motion for robot crawling is 0.31 body length per second. (B) Experimental results and schematic of the robot’s rolling posture. The red line marks the robot’s starting position on the right, and the black line indicates the robot’s termination position on the right. The black arrow signifies the direction of the magnetic field. The normalized speed of motion for robot rolling is 1.88 body length per second. (C) Robot’s rolling motion along the red trajectory within a porcine stomach model. (D) Comprehensive functional validation of the robot’s obstacle overcoming, target grasping, and target transportation capabilities. The red trajectory illustrates the robot’s movement path.

The capability for planar locomotion has been conferred upon the robot through the strategic reconfiguration of its magnetic domain distribution. Validation of the 2 principal locomotive modalities, namely, crawling and rolling, is depicted in Fig. 4A and B and Movie S2. To evaluate the locomotive efficiency of these postures in a manner that is agnostic to the robot’s dimensional disparities, a normalization process was employed, where the displacement of motion was scaled relative to the robot’s body length. When driven at a magnetic field frequency of 1 Hz, the robot achieves a normalized crawling speed of 0.31 body length per second and a normalized rolling speed of 1.88 body length per second, as illustrated in Fig. S1. This demonstrates the robot’s exceptional locomotion capability in the rolling configuration. Nonetheless, given that the rolling motion necessitates a 180-degree alteration of the external magnetic field orientation to complete a cycle, in contrast to the crawling motion, which requires only a 45-degree variation in magnetic field orientation, the latter is deemed more amenable to actuation via a mechanically mounted permanent magnet. Conversely, the rolling motion mandates the incorporation of a rotating magnetic field mechanism for its propulsion.

The precision of the robot’s crawling propulsion is enhanced by shorter effective displacements per cycle, while its rolling motion, requiring less stringent surface morphology, is adept at navigating complex terrains. To substantiate the robot’s applicability in biomedical contexts, a porcine stomach model was engineered and utilized for testing the robot’s rolling capabilities, as illustrated in Fig. 4C and Movie S3. The robot adeptly traversed the model’s inner lining along a predetermined path, thereby validating its ability to sustain motion on surfaces characterized by folds. Additionally, to ascertain the robot’s multifaceted locomotive prowess, a task integrating obstacle negotiation, target acquisition, and transportation was devised, as shown in Fig. 4D and Movie S4. The robot initially surmounted the container’s rim, which was 5 times its thickness, precluding entry via crawling and necessitating a rolling maneuver. Upon reaching the target area, the robot approached red-dyed foam particles, deformed to engage their centers, and adhered them to its pre-glued surface. Subsequently, it relocated to the opposite edge, replicated the rim-crossing action, and exited the area, ultimately retracing its steps to the starting point along a set trajectory to fulfill the designated task. This exercise underscored the robot’s versatile locomotion and its proficiency in overcoming obstacles, directional movement, and the capture and conveyance of objects within intricate settings.

### Aquatic swimming capability of soft robots

**Fig. 5.**
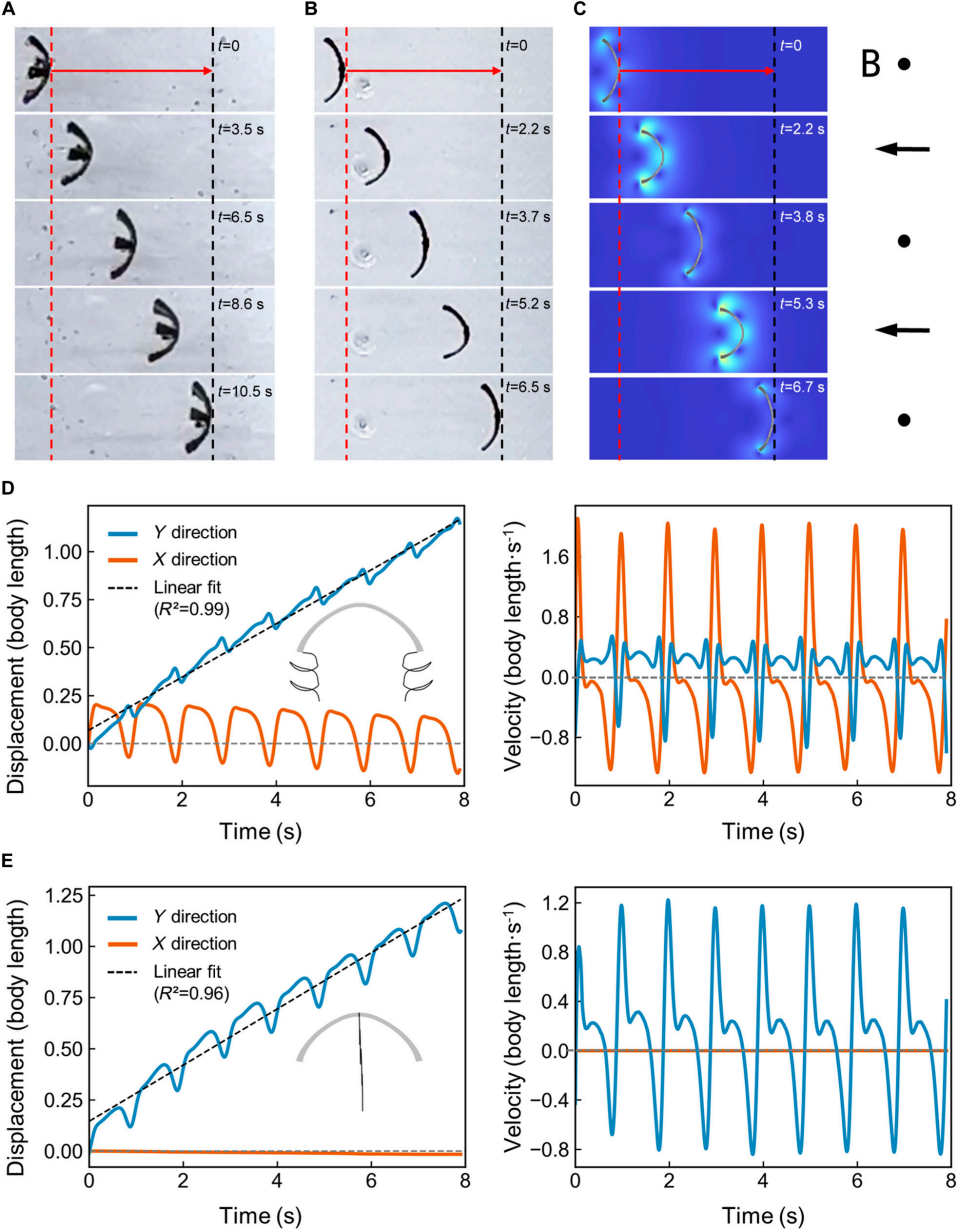
Characterization of the swimming capability of the robot and comparison of experimental and simulation results. (A) Robot’s motion mimicking the jellyfish-like posture in a low Reynolds number fluid. The red line represents the initial right-side position of the robot, and the black line denotes the final right-side position of the robot. The robot swims horizontally to the right. The normalized speed of motion of the robot is 0.14 body length per second. (B) Robot with simplified design, retaining only 2 soft arms, mimicking the jellyfish-like posture in a low Reynolds number fluid. The red line represents the initial right-side position of the robot, and the black line denotes the final right-side position of the robot. (C) Multiphysics coupling simulation results, consistent with the motion posture of the robot with only 2 soft arms. The black arrow indicates the direction of the magnetic field. (D) Displacement–time and velocity–time curves for the left edge point of the soft arm in the simulation results. The gray silhouette shows the motion trajectory of the left edge point of the soft arm in the simulation. (E) Displacement–time and velocity–time curves for the centroid of the robot material in the simulation results. The gray silhouette shows the motion trajectory of the centroid of the material in the simulation.

The robot’s morphological design was inspired by the shape of a jellyfish and is capable of transitioning between expanded and contracted states via magnetic control, enabling it to swim in a liquid environment, as illustrated in Fig. 5A and Movie S5. Due to the minimal resistance in typical liquid environments, observing the propulsion forces associated with structural shape changes is challenging, and the robot’s movement is often affected by gravity, resulting in either floating on the surface or sinking to the bottom. To accurately analyze the robot’s swimming posture in the liquid environment, glycerin with a density of 1.261 g/cm^3^ and a viscosity of 1,499 mPa·s was used, allowing the robot to briefly float and move within the liquid. For a more precise analysis of the robot’s motion, 6 of its soft arms were removed, leaving only 2 symmetrical ones for observation, which also enabled a jellyfish-like swimming posture, as shown in Fig. 5B. The efficiency of the robot’s movement improved by 61% following this modification. This enhancement is likely due to the previous issues with magnetic domain realignment and positional errors during the manual insertion of the robot into the mold. Observations of the robot in its nonideal magnetized state, as depicted in Fig. 5A, revealed rotational movements during swimming, which negatively impacted efficiency. The trimmed robot demonstrated improved performance by addressing these issues.

Based on our previous work on magnetic fluid–solid multiphysics coupling simulations, we have simulated the swimming posture of the robot, as shown in Fig. 5C and Movie S6. In the 2D transient simulation, the robot appears narrower due to the misalignment between the robot’s facing direction and the motion direction during the experimental process. The physical parameters used in the 2D simulation were set according to the parameters of the soft resin with added NdFeB obtained from the experiments, with the overall residual magnetic flux density calculated based on the proportion of NdFeB in the soft resin (37.7 mT). However, the actual material is a complex structure composed of 4 layers, with residual stresses from printing between the layers, resulting in less pronounced deformation under the external magnetic field compared to the simulation results. Nevertheless, within the margin of error, the simulation results are consistent with the experimental outcomes, allowing for further analysis of specific points on the structure using simulation data. To quantitatively assess the robot’s movement capability and exclude the effects of size variations, we normalized the displacement and velocity of its movement by dividing them by the body length.

The robot’s movement primarily relies on the oscillation of the soft arms on both sides. We analyzed the motion speed and displacement of the point on the outermost edge of the soft arm, as shown in Fig. 5D. The *y* direction is defined as the robot’s movement direction, with the positive *y* axis upward, the *x* direction perpendicular to the plane with the positive *x* axis to the right, and the selected point on the outer side of the left soft arm. The simulation reveals that the displacement in the *y* direction of this outer point shows minimal fluctuation, with an excellent linear fit of the displacement–time curve (*R*^2^ = 0.99). During a single cycle, the forward time in the *y* direction is 6.4 times the backward time. However, the displacement and velocity in the *x* direction exhibit marked fluctuations. The robot moves in the *y* direction due to the reciprocating push of the fluid medium under the magnetic field. Since the robot starts from a completely flat, zero-magnetic-field state, the *x* direction displacement of this point is initially positive for the first 2 s. After several cycles of motion, it gradually oscillates around zero displacement. Analysis of a point at the center of the robot’s structure reveals opposite movement patterns in the *x* and *y* directions, as shown in Fig. 5E. The *y* direction displacement of the central point exhibits substantial fluctuations, but the linear fit of the displacement–time curve is good (*R*^2^ = 0.96). The magnetization direction of this point aligns with the driving magnetic field direction, so it is not affected by magnetic torque but reflects the overall movement trend of the robot. The displacement and velocity curves along the *x* direction are more stable compared to those along the *y* direction. Minor displacements in the *x* direction during the simulation could be due to error accumulation. In the calculation process, large nonlinear deformations of the geometric structure can lead to poor mesh quality and calculation failure. To address this, we employed the arbitrary Lagrangian–Eulerian dynamic mesh method, recalculating mesh distortion after each computation and remeshing when distortion exceeded a certain threshold. Remeshing is confined to the free regions defined by boundary conditions. However, changes in the number and position of nodes during each remeshing process can accumulate computational errors, particularly in multiphysics coupling calculations, where deformation of unconstrained soft structures in viscous, low Reynolds number fluid environments may lead to error amplification over time.

## Conclusion

Here, we introduce an advanced DLP technique capable of single-step printing of composite magnetic structures incorporating various materials. This method allows for the integration of soft or rigid resins with different magnetic particles during a single printing cycle, resulting in structures with multifunctional properties. The structures fabricated through this technique demonstrate superior acid and alkali resistance. The cohesion between layers of different materials is notably strong, and the distribution of magnetic particles within homogeneous materials is consistently even. Utilizing this advanced printing technique, we have engineered a soft robotic system that incorporates both hard magnetic and superparamagnetic materials. Our validation efforts have confirmed the programmability of the magnetic domains within the hard magnetic segments of the robot, as well as the heat generation capabilities of superparamagnetic materials when subjected to high-frequency magnetic fields.

Under the influence of a 1-Hz magnetic field, the robotic system can execute diverse locomotive maneuvers with varying velocities: It exhibits a crawling motion at a velocity of 0.31 body length per second, a rolling motion at a velocity of 1.88 body length per second, and a swimming motion at a velocity of 0.14 body length per second. Experimental validation confirmed its capabilities in directed movement, traversing rough surfaces, overcoming obstacles, and capturing and transporting objects. Lastly, experimental and simulation results validated the robot’s swimming ability in high-viscosity, low Reynolds number liquids, with a detailed analysis of the different motion patterns between the soft arm edges and material center points. The 3D printing technology discussed here also holds potential for extension into a broader array of applications, yet it is not without its constraints. The maximum single-layer area of structures printed using current DLP technology is still limited to the range of 500 × 500 mm^2^, a limitation influenced by factors such as the precision of the projection system and the area of the lifting platform. Additionally, the height of the printed structures is hindered not only by the spatial constraints of the printing equipment (a height less than 400 mm) but also by the stability of the magnetic resin over extended printing periods. This issue is anticipated to be addressed through the implementation of a recirculating feed system, among other strategies. Since the magnetic particles currently used do not directly participate in the resin’s reaction, this method could theoretically be expanded to include more magnetic materials that do not react with the resin.

There are several areas in which this printing technology requires further expansion. To enhance the functionality of printed structures, the maximum concentration of hard magnetic particles in magnetic resin limits the magnetic performance of the structures. Further investigation into the curing process of magnetic resin could help address issues such as adhesion of high-concentration magnetic structures to the release film, reduced effective curing depth, and sedimentation of magnetic particles. The development of large-scale printing systems could facilitate the broader application of this technology. At a larger scale, the mechanical properties, magnetic performance, printing precision, and print quality of the structures will require a new comprehensive assessment, and a new set of performance evaluation standards will need to be established. In terms of materials, the development of new high-performance magnetic materials with low biological toxicity could substantially expand the bioapplication prospects of printed structures. In terms of specific applications, the reliability of printed robots within ex vivo biological organs needs to be verified. This work also serves to test the biocompatibility of new research materials. Specifically, we plan to design capsule robots with drug delivery capabilities to transport drug particles to the wound sites of biological tissues. Additionally, we aim to develop swimming robots with a layer of superparamagnetic material that can navigate to designated locations to generate heat and kill cancer cells.

Overall, the multimaterial 3D printing technology proposed in this study shows great promise in the design and fabrication of multifunctional soft robots. This technology could inspire the design and fabrication of new multifunctional and multimaterial soft robots.

## Data Availability

All data included in this study are available upon request by contacting the corresponding author.
