# Supplementary material for: Enhanced Digital Light Processing-Based One-Step 3-Dimensional Printing of Multifunctional Magnetic Soft Robot
Source: Cyborg Bionic Syst. 2025 Feb 26;6:0215. doi: 10.34133/cbsystems.0215 (PMC11861425; doi:10.34133/cbsystems.0215)
Supplement: Supplementary 1 — Figs. S1 to S3 Movies S1 to S6 [file cbsystems.0215.f1.zip › cbsystems.0215.f1.docx]

Supporting Information

**Enhanced DLP-Based One-Step 3D Printing of Multifunctional Magnetic Soft Robot**

Zhaoxin Li^1^, Ding Weng^1^, Lei Chen^1^, Yuan Ma^1^, Zili Wang^1^, Jiadao Wang^1*^


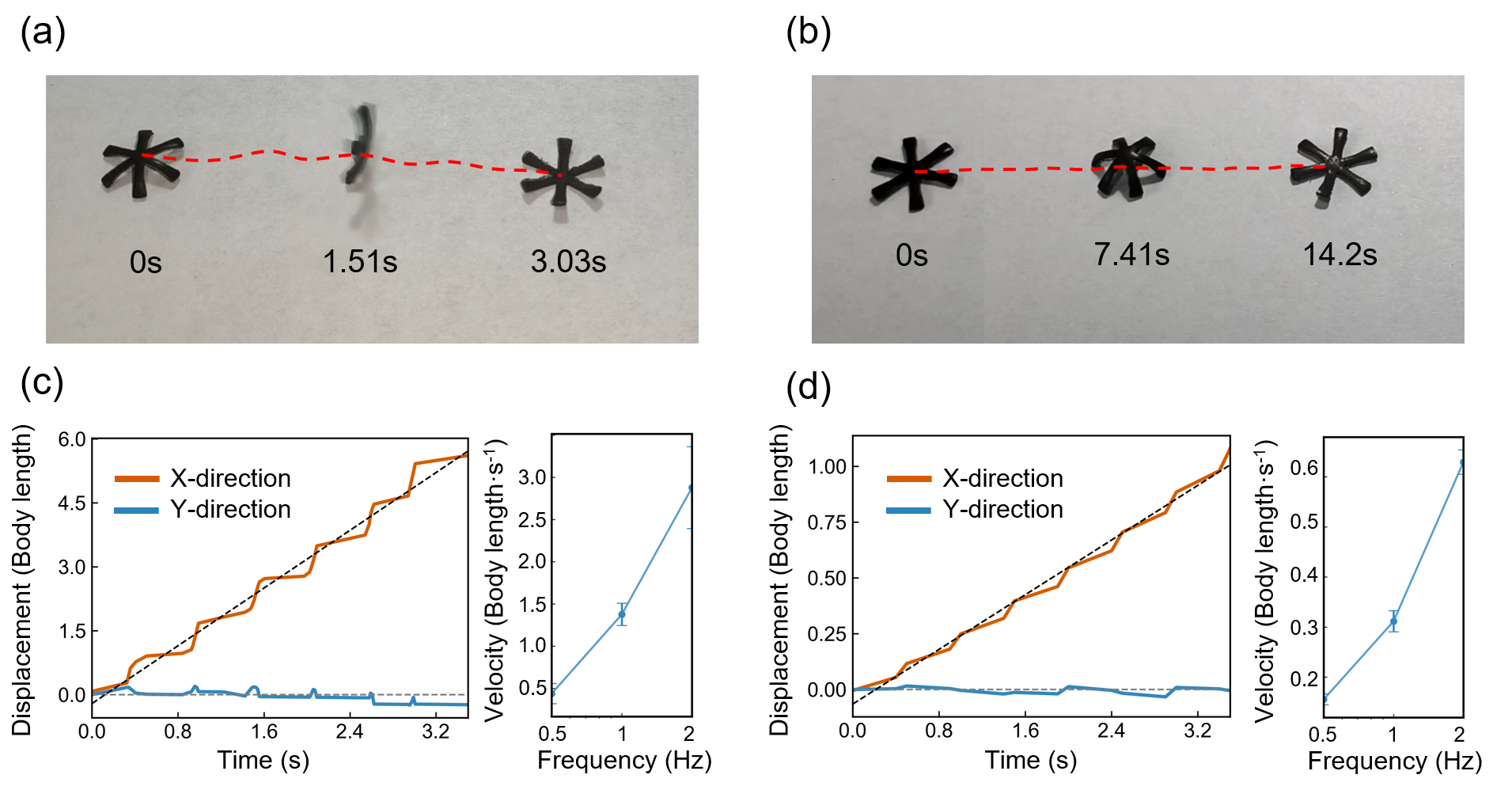


Figure S1. Performance Metrics of the Robot in Rolling and Crawling States. (a) and (b) Experimental schematic illustrations of the robot's rolling and crawling processes, with the red dashed lines indicating the trajectory of the robot's center of mass. Specific timestamps are marked at intermediate positions along the trajectory. (c) and (d) Displacement-time and velocity-magnetic frequency curves for the robot in rolling and crawling states. Dashed lines represent the linear fit of displacement along the X-axis and the initial position along the Y-axis.


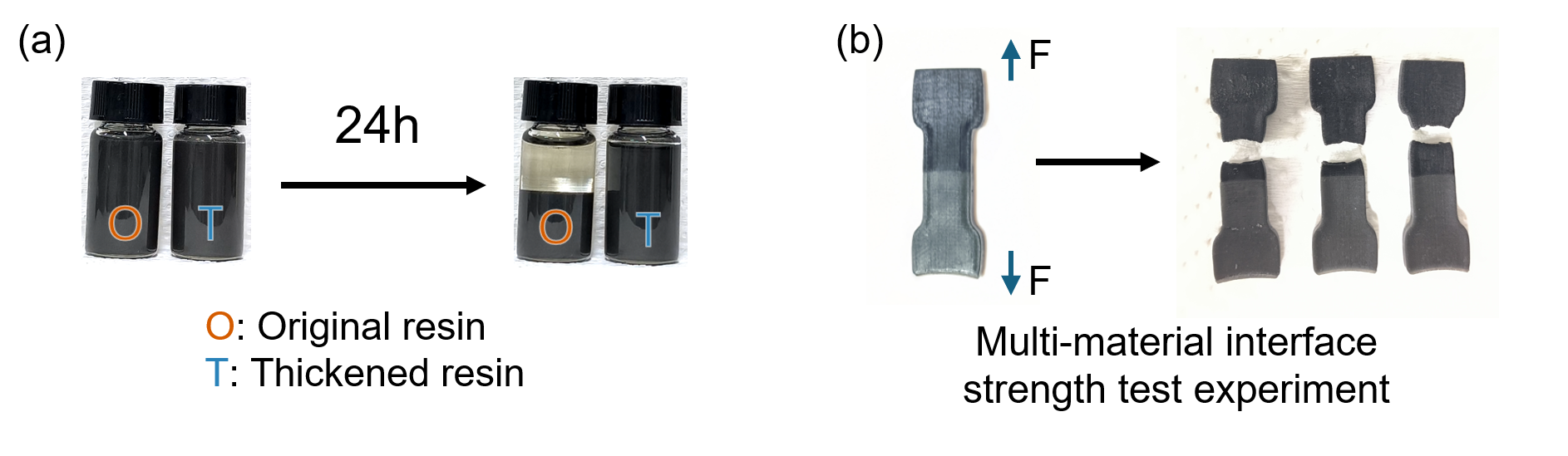


Figure S2. Assessment of resin stability and interfacial strength in multi-material interfaces. (a) The untreated magnetic resin demonstrated significant sedimentation, with over 50% of its volume becoming transparent after a 24-hour period. Conversely, the thickened magnetic resin maintained stability, with a sedimentation rate of less than 5% over the same duration. (b) Tensile testing was performed on 3D-printed samples of magnetic multi-materials to ascertain interfacial strength. The structure was bifurcated, with the upper section incorporating nanoscale Fe3O4 particles and the lower section containing microscale NdFeB particles. The samples consistently failed at the interface rich in nanoscale Fe3O4 particles, presenting a retracted fracture surface upon failure.


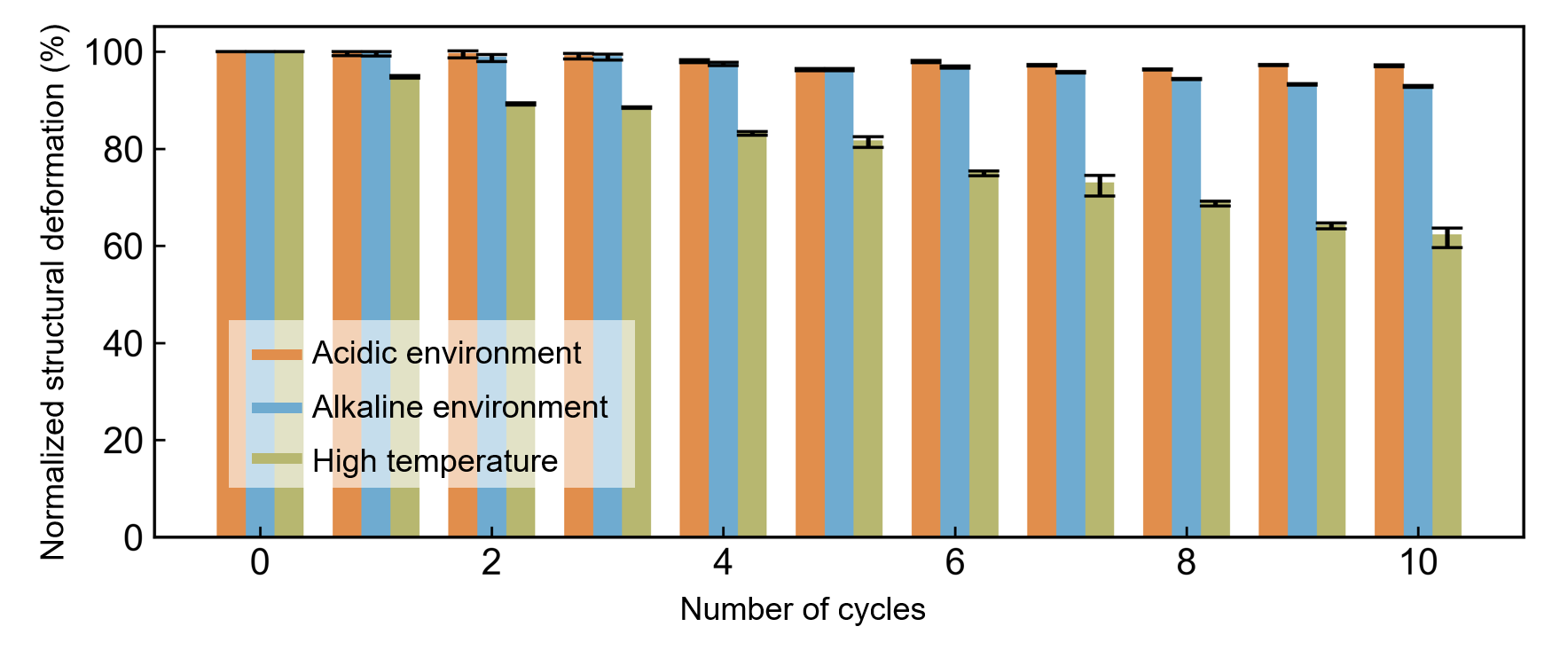


Figure S3. The variation in structural deformation capability of 3D-printed cantilever beam structures under acidic, alkaline environments, and high temperatures as a function of cycling number. The acidic environment is defined by an artificial gastric fluid at pH=1.5, the alkaline environment by a NaOH solution at pH=14, and the high-temperature environment by a setting at 100°C. During each cycle, the cantilever beam is immersed in the respective environment for one hour before being removed. Post each cycle, the cantilever beam structure is placed within a uniform magnetic field to measure the deflection angle, with results normalized for consistency.
